# Development of Simplified Models for Non-Destructive Hyperspectral Imaging Monitoring of S-ovalbumin Content in Eggs during Storage

**DOI:** 10.3390/foods11142024

**Published:** 2022-07-08

**Authors:** Kunshan Yao, Jun Sun, Jiehong Cheng, Min Xu, Chen Chen, Xin Zhou, Chunxia Dai

**Affiliations:** 1 School of Electrical and Information Engineering, Jiangsu University, Zhenjiang 212013, China; yks1994@sina.com (K.Y.); cheng_jeho@sina.com (J.C.); minxu1987415@sina.com (M.X.); zhouxin_21@ujs.edu.cn (X.Z.); txdcx@126.com (C.D.); 2 School of Economics and Management, Jiangsu University of Science and Technology, Zhenjiang 212100, China; fmgxiaochen@sina.com

**Keywords:** hyperspectral imaging, egg, S-ovalbumin content, visualization, multivariate analysis

## Abstract

S-ovalbumin content is an indicator of egg freshness and has an important impact on the quality of processed foods. The objective of this study is to develop simplified models for monitoring the S-ovalbumin content of eggs during storage using hyperspectral imaging (HSI) and multivariate analysis. The hyperspectral images of egg samples at different storage periods were collected in the wavelength range of 401–1002 nm, and the reference S-ovalbumin content was determined by spectrophotometry. The standard normal variate (SNV) was employed to preprocess the raw spectral data. To simplify the calibration models, competitive adaptive reweighted sampling (CARS) was applied to select feature wavelengths from the whole spectral range. Based on the full and feature wavelengths, partial least squares regression (PLSR) and least squares support vector machine (LSSVM) models were developed, in which the simplified LSSVM model yielded the best performance with a coefficient of determination for prediction (R^2^_P_) of 0.918 and a root mean square error for prediction (RMSEP) of 7.215%. By transferring the quantitative model to the pixels of hyperspectral images, the visualizing distribution maps were generated, providing an intuitive and comprehensive evaluation for the S-ovalbumin content of eggs, which helps to understand the conversion of ovalbumin into S-ovalbumin during storage. The results provided the possibility of implementing a multispectral imaging technique for online monitoring the S-ovalbumin content of eggs.

## 1. Introduction

Eggs are a staple food for humans and are rich in nutrients. Due to the excellent gel forming and foaming properties of albumin, eggs are widely used in many food prod-ucts [1,2]. The albumen contains a variety of proteins, including ovalbumin, ovotrans-ferrin, ovomucoid, lysozyme, and globulins; among these, ovalbumin contains free sulfhydryl groups [3]. During storage, ovalbumin is naturally and irreversibly converted into a thermally stable form, S-ovalbumin [4]. The rate of conversion is only related to albumen pH and temperature, not to the age of the egg, variety, or nutritional status; thus, S-ovalbumin content has been developed as an indicator to evaluate the freshness of eggs [5]. 

The denaturation temperature of ovalbumin is approximately 80 °C [6]. During heating, the sulfhydryl groups in ovalbumin are released and converted into disulfide bonds, which play an important role in the formation of protein networks and the properties of albumen gels [7,8]. Compared with ovalbumin, S-ovalbumin has a relatively higher thermal stability with a denaturation temperature of 88 °C [6]. As a result, the disulfide bonds are formed later in S-ovalbumin. This difference can lead to a decrease in the strength of the heated-induced gel, thus affecting the quality of processed foods [8]. For instance, the content of S-ovalbumin has a significant impact on the cohesiveness and springiness of cake crumbs [8] and the extensibility of noodles [9]. Therefore, the detection of S-ovalbumin content in eggs is critical to the food processing industry.

At present, the content of S-ovalbumin is mainly determined by spectrophotometry [5]. However, chemical testing is destructive and time-consuming, and is gradually failing to meet the needs of the industry [10]. Therefore, it is desired to develop a non-invasive and rapid method to detect the S-ovalbumin content in eggs. Previous investigations on the non-destructive evaluation of egg qualities have used visible–near infrared spectroscopy [11], machine vision [12], electronic nose [13], and acoustic resonance [14]. However, the aforementioned methods have their own merits and drawbacks. For example, machine vision cannot offer information associated with the chemical composition [15]. In addition, neither spectroscopy, electronic nose, nor acoustic resonance can obtain the spatial distribution information [16]. To overcome these defects, a unique imaging technique is needed to map the location of each tested component, and this requirement can be well satisfied by hyperspectral imaging (HSI) [17,18]. HSI integrates traditional imaging and spectroscopy techniques, which can synchronously obtain the spatial and spectral information of the target [19,20]. In terms of egg quality assessment, HSI has been successfully used for the detection of the Haugh unit (HU), albumen pH, bubbles, scattered yolk, and blood spots [21,22,23,24]. However, research on the detection and visualization of S-ovalbumin content in eggs has not been reported in the literature.

Therefore, this study aims to develop a non-destructive method to monitor the S-ovalbumin content in eggs during storage based on HSI and multivariate analysis.

## 2. Materials and Methods

### 2.1. Egg Samples

A total of 180 fresh eggs were obtained from a poultry farm in Zhenjiang, China, and stored at 25 °C (55% relative humidity). Eighteen eggs were randomly selected for measurement after storage for 0, 3, 6, 9, 12, 15, 18, 21, 24 and 27 days, respectively.

### 2.2. Hyperspectral Images Acquisition and Calibration

**Figure 1:**
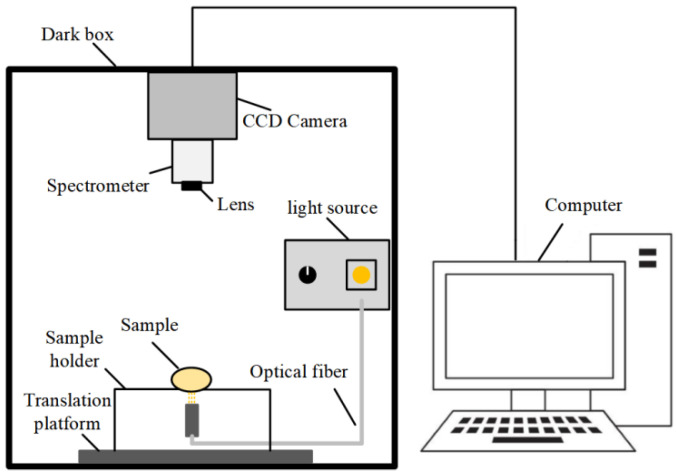
Hyperspectral imaging system.

The visible–near infrared (VIS–NIR) HSI system is shown in Figure 1, which has been described in detail in our recent research [25]. The egg sample was horizontally placed on the translation platform and scanned in the transmission mode with a spectral range of 401–1002 nm. The moving speed of the translation platform was fixed to 1 mm/s to avoid overlapping frames, and the CCD camera exposure time was set to 0.1 s. Each hyperspectral image is a 3-D data cube that contains 478 images with different wavelengths, saved in raw format.

To reduce the influence of dark current in camera, the acquired hyperspectral images were further calibrated by:

(1) $$I_{c}=\frac{I_{o}-I_{d}}{I_{w}-I_{d}}$$

where *I_c_* indicates the calibrated image, *I_o_* represents the raw image, *I_w_* is the white reference image (~99% transmittance) obtained with a standard white board, and *I_d_* is the dark reference image (~0% transmittance) obtained by covering the camera lens.

### 2.3. Determination of S-ovalbumin

In this study, the reference for S-ovalbumin content of eggs was measured by spectrophotometry [5]. Five grams of albumen was firstly taken into a 100 mL beaker, mixed with 25 mL of 0.5 mol/L phosphate buffer and magnetically stirred for 5 min. Next, 5 mL of the suspension was placed into a test tube and heated in a water bath pot at 75 °C for 30 min. Upon cooling, 10 mL of the precipitating solution was added and centrifuged at 12,000 rpm for 5 min. Then, 2 mL of the supernatant was placed into a test tube, mixed with 4 mL of the biuret solution. After standing for 30 min, the absorbance at 540 nm was determined using an ultraviolet spectrophotometer (TU-1810, Purekinje General Instrument Ltd., Beijing, China), denoted as *O_heated_*. In addition to water bath heating, the above steps were repeated to obtain an absorbance of *O_unheated_*. The content of S-ovalbumin was calculated as follows:

(2) $$S-ovalbu\min(\%)=\frac{O_{heated}}{O_{unheated}}\times 100\%$$

### 2.4. Spectral Data Extraction

**Figure 2:**
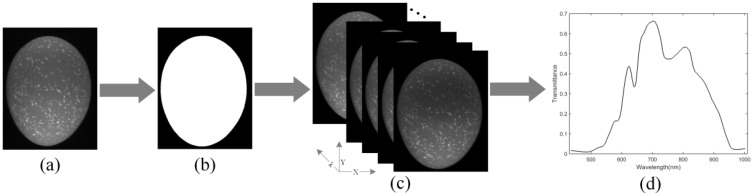
Spectral data extraction of ROI (**a**) Image at 700.4 nm; (**b**) binary mask image; (**c**) result of ROI identification; (**d**) mean spectral curve of ROI.

After calibrating the hyperspectral image, the region of interest (ROI) can be easily identified because of the significant difference in transmitted light intensity between the egg sample and the background. Therefore, a simple threshold segmentation method was applied to extract the spectrum of ROI, as shown in Figure 2. The high transmission wavelength image at 700.4 nm (Figure 2a) was first employed to generate a binary mask image (Figure 2b) by setting a transmittance threshold of 0.1. By applying the mask image, the ROI and background in each single wavelength image were separated, and the pixel value of background was set to 0 (Figure 2c). Finally, the mean value of the pixels in ROI was calculated as the transmission spectrum of egg sample, as shown in Figure 2d. Due to the low signal-to-noise ratio of the hyperspectral images before 435 nm, no useful information can be extracted from this region. Thus, the final adopted effective spectral range was 435–1002 nm with a total of 449 wavelengths.

### 2.5. Spectral Pre-Prcossing

The egg samples were divided into a calibration set (2/3) and a prediction set (1/3) by using sample set partitioning based on the joint X-Y distances (SPXY) algorithm [26]. The spectral data contain a large amount of useful information related to S-ovalbumin, as well as some redundant and interfering information. To improve the modeling accuracy and robustness, the standard normal variate (SNV) was used for spectral pre-processing, which can remove the influence of undesirable systematic, scattering and light distance changes [27]. The formula of SNV is as follows:

(3) $$X_{cor}=\frac{X_{org}-\bar{X}}{\sqrt{\sum_{1}^{N}{\left(X_{org}-\bar{X}\right)}^{2}/(N-1)}}$$

where *X_org_* and *X_cor_* represent the original and corrected sample spectrum, respectively; $\bar{X}$ indicates the mean value of the spectrum, and *N* is the number of wavelengths.

### 2.6. Modeling

Selecting robust and reliable multivariate analysis methods to establish calibration models is of great importance for quantitative analysis. In this study, partial least squares regression (PLSR) and least squares support vector machine (LSSVM) were used to correlate the spectral data with the reference S-ovalbumin content values measured by spectrophotometry.

#### 2.6.1. PLSR

PLSR is a classical linear regression method, which has been widely applied to build a prediction model between the spectral response and the quality index [28]. The regression function of PLRS is [29]:

(4) Y=Xb+E

where *X*_(*n*×*p*)_ is the input matrix, *Y*_(*n*×1)_ is the output matrix, *b* is the regression coefficient matrix and *E* is the residual error matrix. In PLSR, the high-dimensional data is projected onto a small number of latent variables (LVs) to find the optimal regression coefficients so that a linear combination of the input variables maximizes the covariance between the LVs and the output. When one supposes that the number of LVs is *h* (*h* ≦ *p*), the regression coefficients are calculated as follows [30]:

Step 1: Calculate the loading weights: *w = X^T^Y*;

Step 2: Normalize the loading weights: *w = w/||w||*;

Step 3: Calculate the score of *X*: *S = Xw*, and the loading vectors of *X* and *Y*: *p* = *X^T^s/(s^T^s)*, *q* = *Y^T^s/(s^T^s)*;

Step 4: Save *w*, *s*, *p* and *q* in *W*, *S*, *P* and *Q*, respectively;

Step 5: Deflate *X* and *Y*: *X* = *X* − *sp^T^, Y* = *Y* − *sq*;

Step 6: Repeat Steps 1–5 for the *h* LVs;

Step 7: The regression coefficients can be calculated by: *b = W^T^* (*PW^T^*)^−1^ *Q*.

#### 2.6.2. LSSVM

LSSVM is developed on the basis of SVM, which transforms inequality constraints into equality constraints and replaces quadratic programming with least square cost function [31]. When dealing with the problem of non-linear function approximation, LSSVM has the advantages of simple computation, fast solution speed and strong generalization [32]. The model structure of LSSVM is:

(5) $$f(x)=w^{T}\phi (x)+b$$

where *φ* (*x*) is the mapping function, and the weight vector *w* and bias vector *b* are obtained by minimizing the regularized risk function of LSSVM.

(6) $$\begin{array}{l} \min\;\frac{1}{2}w^{T}w+\frac{1}{2}\gamma \sum_{i=1}^{l}\xi_{i}^{2}\\ s.t.\;y_{i}=w^{T}\phi (x_{i})+b+\xi_{i},\;i=1,\;2,\;\ldots ,\;l \end{array}$$

where *ξ_i_* is the slack variable and *γ* is the regularization parameter. By applying the Lagrangian function and the KKT condition, Equation (5) can be transformed into:

(7) $$f(x)=\sum_{i=1}^{l}\alpha_{i}K(x,x_{i})+b$$

where *α_i_* is the Lagrangian multiplier and *K* (*) is the kernel function that satisfies the Mercer condition. In this study, a radial basis function (RBF) kernel was used, which can not only achieve non-linear mapping, but also has the advantage of fewer parameters [33], as shown in Equation (8):

(8) $$K(x,x_{i})=\exp(-||x-x_{i}|{|}^{2}/2\sigma^{2})$$

where *σ* is the kernel width.

The performance of the established models was statistically assessed using the determination coefficient and the root mean squared errors for calibration (R^2^_C_, RMSEC), cross-validation (R^2^_CV_, RMSECV) and prediction (R^2^_P_, RMSEP). In general, a reliable model should have lower values of RMSEC, RMSECV and RMSEP and higher values of R^2^_C_, R^2^_CV_ and R^2^_P_, and the difference between them should be small [34]. The operation of PLSR and LSSVM was carried out in Matlab R2014b.

### 2.7. Feature Wavelength Selection

Due to the wide spectral range of HSI, multicollinearity and redundancy among adjacent wavelengths are common in hyperspectral data. To simplify the calibration models and improve the detection efficiency, it is necessary and crucial to select the feature wavelengths from the whole spectral range [35]. In the current study, competitive adaptive reweighted sampling (CARS) was applied to optimize the wavelengths for S-ovalbumin content prediction. CARS is a relatively novel feature variable selection method, which integrates monte carlo (MC) sampling, exponentially decreasing function (EDF), adaptive reweighted sampling (ARS) and partial least squares (PLS) techniques [36]. The steps of CARS are as follows:

Step 1: A certain proportion of samples are selected by MC sampling to build a PLS model, and the absolute value of the regression coefficient is taken as the weight of each variable.

Step 2: EDF is applied to remove the variables with small weights, and then ARS that simulates the principle of ‘survival of the fittest’ and is used to further select key variables.

Step 3: The key variables are used to rebuild a PLS model, and the RMSECV of the new model is calculated. In addition, the key variables are utilized to generate initial samples in the next iteration.

Step 4: Steps 1–3 are alternated until the predefined number of MC sampling is reached, and the variables in the PLS model with the minimum RMSECV are identified as feature variables.

### 2.8. Visualization of S-ovalbumin Content

Visualizing the distribution of S-ovalbumin can help to understand the conversion of ovalbumin into S-ovalbumin in eggs during storage. With the help of HSI, the predictive attributes can be visualized in a pixel manner by inputting spectral data into the calibration model [37]. In this study, the optimal model was utilized to predict the S-ovalbumin content of each pixel in hyperspectral images, and then the generated chemical images were displayed with a linear color bar. By observing the color variation in chemical images, one can intuitively evaluate the S-ovalbumin content and freshness of eggs, which facilitates the industry in storage temperature regulation and inventory management.

## 3. Results and Discussion

### 3.1. S-ovalbumin Analysis

**Figure 3:**
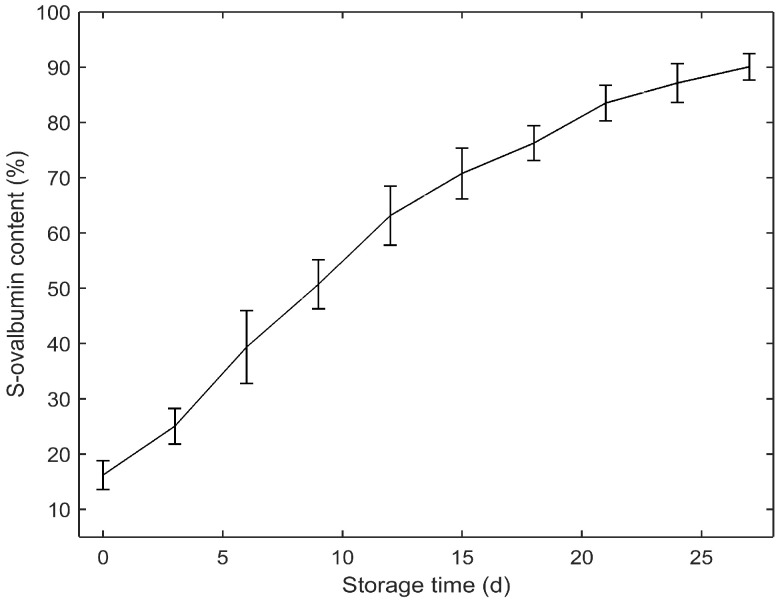
S-ovalbumin content of egg samples at different storage periods.

The statistics of S-ovalbumin content in egg samples measured by spectrophotometry are presented in Table 1. The S-ovalbumin content varied widely between 10.95% and 94.48%, which was essential for the development of robust calibration models. Figure 3 shows the mean S-ovalbumin content of eggs at different storage periods. An evidently increased tendency over storage time can be observed, which is in agreement with the report of Huang et al. [5].

### 3.2. Spectral Feature Analysis

**Figure 4:**
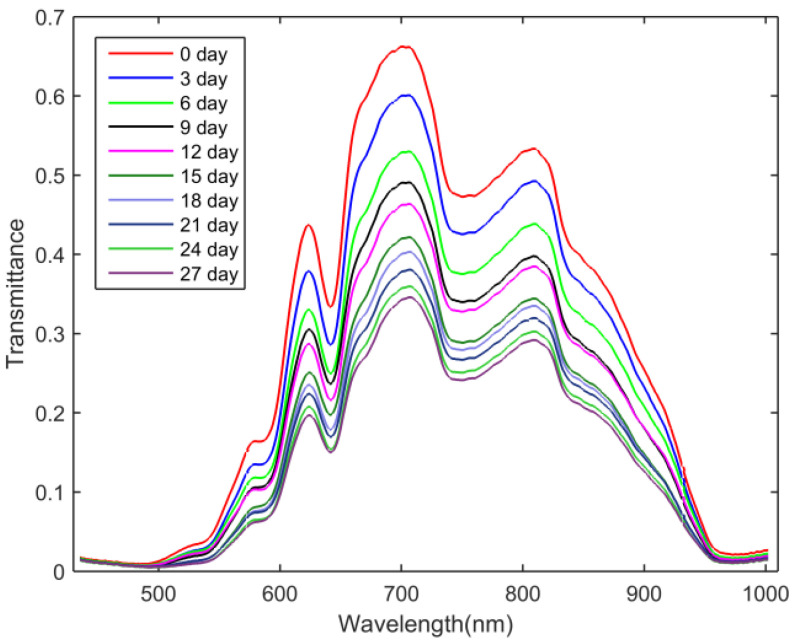
Mean transmittance spectra of egg samples at different storage times.

Figure 4 shows the mean spectral curves (435–1002 nm) of egg samples stored for 0, 3, 6, 9, 12, 15, 18, 21, 24 and 27 days. With the increase in storage time, a decreasing trend can be observed from the mean transmission spectra of egg samples, however, the general shapes and trends of the spectral curves were similar. As seen from Figure 4, the main absorption bands of eggs were identified at around 589, 643, 750 and 970 nm. The absorption bands at 589 and 643 nm were attributed to the pigment protoporphyrin in the eggshell [38,39], and the bands at around 750 and 970 nm were related to the third and second overtone of O-H stretching, respectively [40,41].

**Figure 5:**
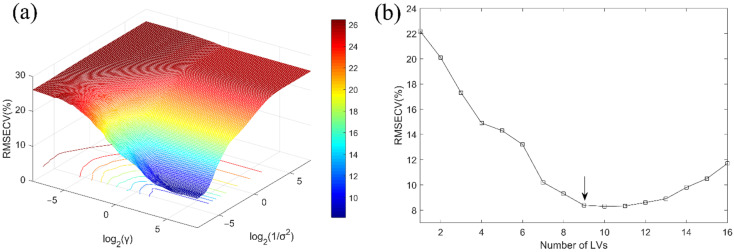
(**a**) RMSECV plot for identifying the optimum γ and 1/σ^2^ in LSSVM model, (**b**) The changing trend of RMSECV with the increase of LVs in PLSR model.

To correlate the spectra with the reference S-ovalbumin content values, the calibration models were developed using PLSR and LSSVM based on the full wavelengths. Selecting an appropriate regularization parameter (γ) and kernel parameter (1/σ^2^) is crucial for LSSVM to avoid over-fitting or under-fitting and to improve modeling ac-curacy [42]. Therefore, five-fold cross-validation was used to estimate the optimal γ and 1/σ^2^. In addition, the optimum number of LVs in PLSR model was evaluated using the same approach. Figure 5 shows the parameter selection process for LSSVM and PLSR models, where the optimization ranges of γ, 1/σ^2^ and LVs were set to [2^−^^8^, 2^8^], [2^−^^8^, 2^8^] and [1, 16], respectively. Figure 5a shows the 3D view of the RMSECV for the LSSVM model with different values of γ and 1/σ^2^. When the combination of log2(γ) and log2(1/σ^2^) was [5.9, -7.9], the RMSECV of the established LSSVM model achieved the minimum. It can be seen from Figure 5b that, as the number of LVs increased, the RMSECV of the developed PLSR model first decreased sharply and then increased slowly. When the number of LVs was greater than 9, the decrease in RMSECV was no longer significant. Therefore, the number of LVs in the PLSR model for predicting S-ovalbumin content was set to 9. Table 2 shows the statistics used for evaluating the performance of models, in which LSSVM exhibits a better performance (R^2^_P_ = 0.893, RMSEP = 8.165%), proving the feasibility of applying HSI in the prediction of S-ovalbumin content.

### 3.3. Prediction of S-ovalbumin Content Based on Feature Wavelengths

**Figure 6:**
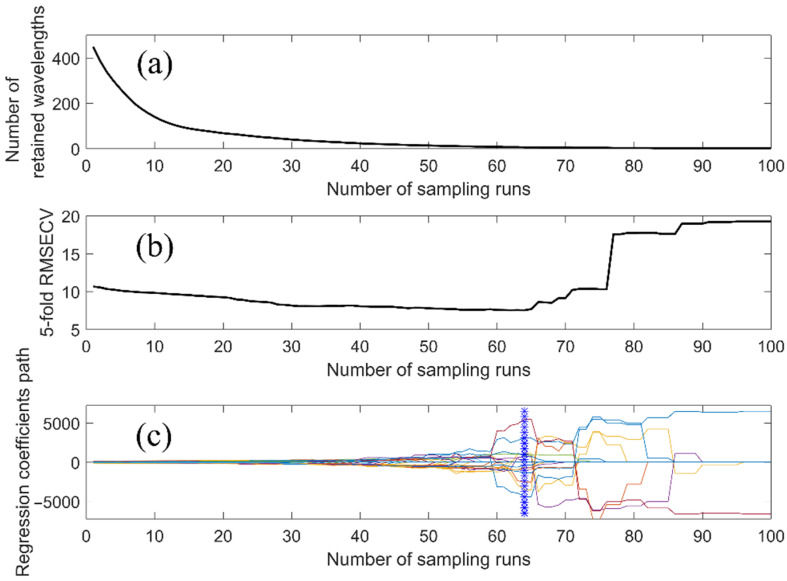
The process of feature wavelength selection by CARS. (**a**) The number of retained wavelengths with the increase in sampling runs; (**b**) the 5-fold RMESCV of the PLS model established based on the retained wavelengths; (**c**) the regression coefficients of the retained wavelengths in the PLS model.

Feature wavelength selection can effectively eliminate the overlapping and redundant information in spectral data, and is a key procedure for developing an on-line multispectral imaging system with fewer wavelengths. In this research, CARS was used to select feature wavelengths aiming to develop simplified models, where the number of MC sampling was set to 100 and five-fold cross-validation was utilized. The process of feature wavelength selection by CARS is shown in Figure 6. Figure 6a shows the number of retained wavelengths with the increase in sampling runs, Figure 6b shows the five-fold RMESCV of the PLS model established based on the retained wavelengths and Figure 6c shows the regression coefficients of the retained wavelengths in the PLS model. From Figure 6a, it can be seen that the wavelength selection process can be divided into two stages: fast selection (sampling runs 1–30) and refined selection (sampling runs 31–100). In the fast selection stage, most of the Interfering and overlapping wavelengths were rapidly removed, resulting in a fast reduction in RMSECV. In the refined selection stage, the wavelengths with little or no information were eliminated in a stepwise manner, leading to a slow change in RMSECV from sampling runs 31–64, followed by a rapid increase due to the elimination of some informative wavelengths from the optimal subset (denoted by blue *). Finally, fourteen wavelengths, 615.5, 624.3, 680.0, 687.6, 700.4, 711.8, 734.9, 758.0, 787.6, 837.9, 870.3, 896.2, 953.1, and 963.3 nm were selected by CARS. The feature wavelengths were mainly located at around 620, 700, 740, 836, 880, and 960 nm, which were assigned as follows: electron transitions of lipid; pH and pigment molecule in albumen; the third overtone of O-H in water; the combinations and overtone modes of water vibration; the stretching overtones of N-H and C-H related to protein and lipid; and the second overtone of O-H in water, respectively [34,40,41]. Half of the selected wavelengths were close to those reported by Zhang et al. [21], whose research indicated that the wavelengths related to the changes of egg freshness were 620, 632, 654, 671, 680, 684, 697, 707, 712, 724, 762, 780, and 796 nm, which could explain the finding of Huang et al. [5] that HU is highly correlated with S-ovalbumin content from the perspective of spectral analysis.

**Figure 7:**
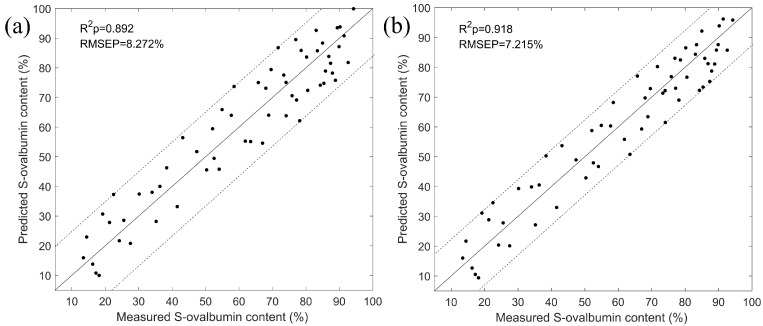
Predicted and measured S-ovalbumin content values for (**a**) PLSR and (**b**) LSSVM models based on feature wavelengths.

Based on the fourteen feature wavelengths, the simplified LSSVM and PLSR models were established to predict the S-ovalbumin content of eggs, and the statistical results are shown in Table 2. It can be seen that the performance of the simplified models was significantly better than the models based on full wavelengths. The improvement is due to the fact that CARS removes redundant wavelengths while retaining as much feature-related information as possible. In addition, it was noticed that LSSVM had better performance than PLSR whether based on the full wavelengths or the feature wavelengths, indicating that the non-linear regression model is more suitable for detecting the S-ovalbumin content of eggs. To graphically display the performance of the simplified LSSVM and PLSR models, the measured and predicted S-ovalbumin content values are plotted and shown in Figure 7. Consequently, the values of R^2^_p_ and RMSEP of the simplified LSSVM model were 0.918 and 7.215%, respectively. Therefore, HSI combined with the simplified LSSVM model can be effective for the detection of S-ovalbumin content in eggs.

### 3.4. Visualization of S-ovalbumin Contents

**Figure 8:**
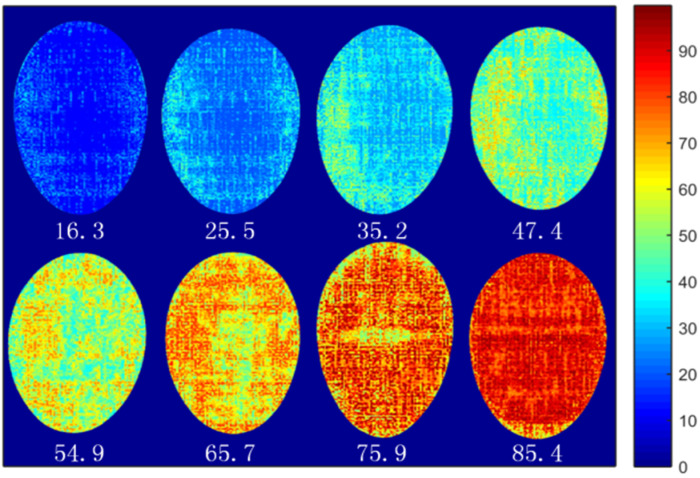
Visualization of S-ovalbumin contents in eggs.

The LSSVM model based on the optimized wavelengths was applied to predict the S-ovalbumin content of each pixel in hyperspectral images. Figure 8 shows the chemical maps of egg samples stored for different times. The pixels with higher S-ovalbumin content were displayed in red, while the pixels with lower S-ovalbumin content were shown in blue. With the increase in S-ovalbumin content, the color of the chemical map has an evident change in trends. When the S-ovalbumin content reached 85.4%, the whole egg was nearly red. The chemical maps showed irregular colors in various parts of eggs, indicating that the distribution of S-ovalbumin in eggs was uneven. This result is consistent with the report of Suktanarak et al. [43] whose study indicated that the internal freshness and albumen quality of eggs are non-homogeneous. According to the color of chemical maps, the S-ovalbumin content and freshness of eggs can be evaluated intuitively. In practical application, when the S-ovalbumin distribution does not meet the criteria, processors can adjust the storage temperature.

## 4. Conclusions

In this work, HSI was applied to monitor the S-ovalbumin content of eggs. The mean transmission spectra of eggs decreased with the increase in S-ovalbumin content. The LSSVM and PLSR models developed on the full spectral wavelengths achieved good performance in S-ovalbumin content prediction (LSSVM: R^2^_P_=0.893, RMSEP=8.165%; PLSR: R^2^_P_=0.861, RMSEP=9.494%). After feature wavelength selection, the performance of LSSVM and PLSR models improved and the simplified LSSVM model yielded the best result with R^2^_P_ of 0.918 and RMSEP of 7.215%. By transferring the simplified LSSVM model to the hyperspectral images, the distribution maps were generated to visualize the S-ovalbumin content. The result indicates that HSI is promising in monitoring the S-ovalbumin content in eggs during storage.

## Figures and Tables

**Table 1:** Reference S-ovalbumin content of egg samples measured by spectrophotometry.

Indexes	Calibration Set	Prediction Set
Number of samples	120	60
Minimum (%)	10.95	13.45
Maximum (%)	94.48	94.24
Mean (%)	61.05	60.28
Standard deviation (%)	26.02	25.40
Range (%)	83.53	80.79

**Table 2:** Performance of models for predicting S-ovalbumin content in eggs.

Model	Variable Number	Calibration		Cross-Validation		Prediction	
		R^2^_C_	RMSEC(%)	R^2^_CV_	RMSECV(%)	R^2^_P_	RMSEP(%)
LSSVM	449	0.943	6.121	0.920	7.238	0.893	8.165
PLSR	449	0.929	6.685	0.899	8.025	0.861	9.494
LSSVM	14	0.952	5.604	0.929	7.068	0.918	7.215
PLSR	14	0.941	6.395	0.912	7.585	0.892	8.272

## Data Availability

The data used and/or analyzed during the current study are available from the corresponding author on reasonable request.
